# Supplementary material for: Frequency-selective perovskite photodetector for anti-interference optical communications
Source: Nat Commun. 2024 Mar 7;15:2066. doi: 10.1038/s41467-024-46468-5 (PMC10920912; doi:10.1038/s41467-024-46468-5)
Supplement: Supplementary file 1 — Supplementary Information [file 41467_2024_46468_MOESM1_ESM.pdf]

Supplementary Information

Frequency-selective perovskite photodetector for anti-interference optical communications

Min et al.

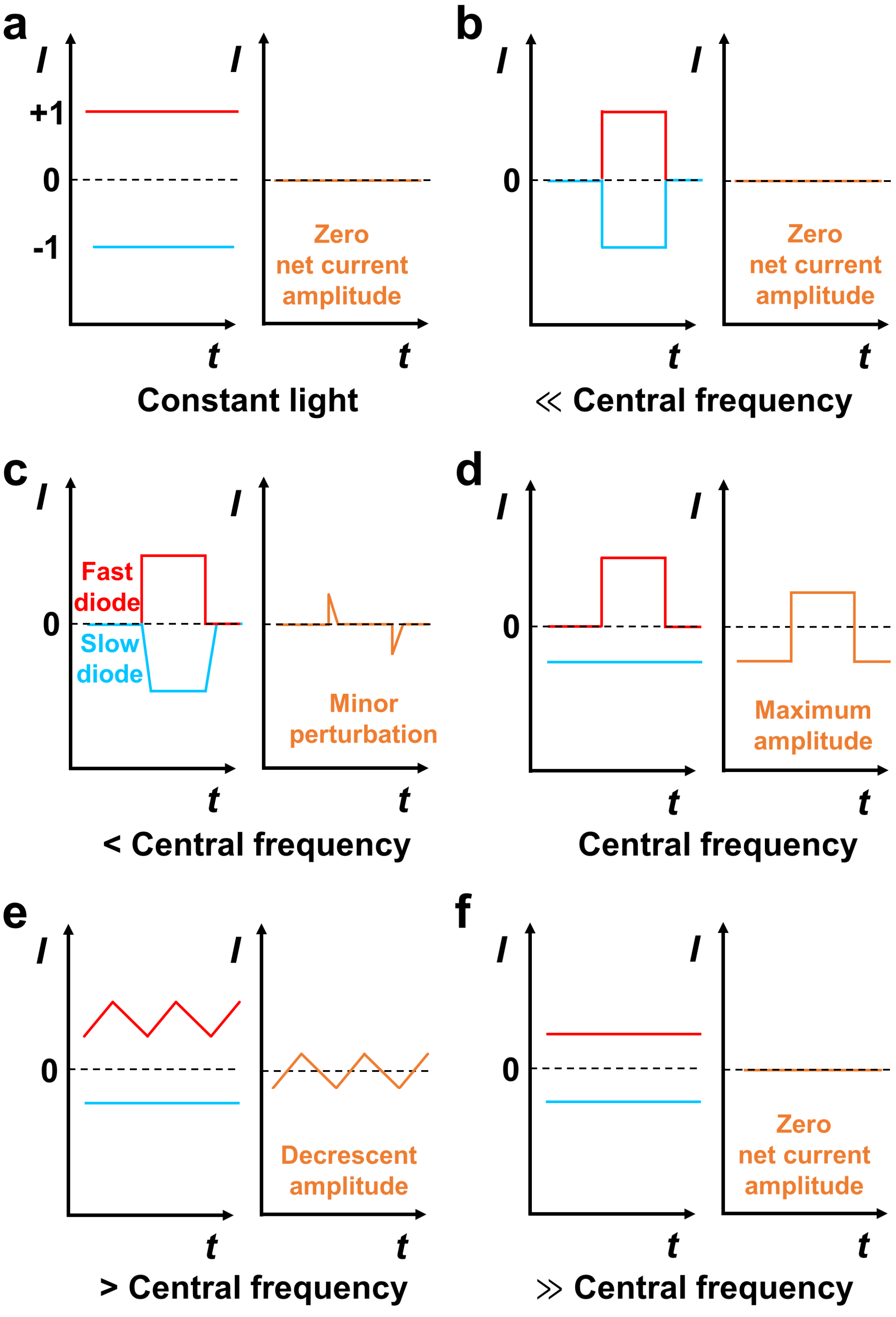

Supplementary Figure 1 | Design principles of the devices. a-f, Diagrammatic sketch of the currents from the two back-to-back photodiodes under constant light (**a**), far less than central frequency (**b**), less than central frequency (**c**), central frequency (**d**), greater than central frequency (**e**), and far greater than central frequency (**f**) conditions.

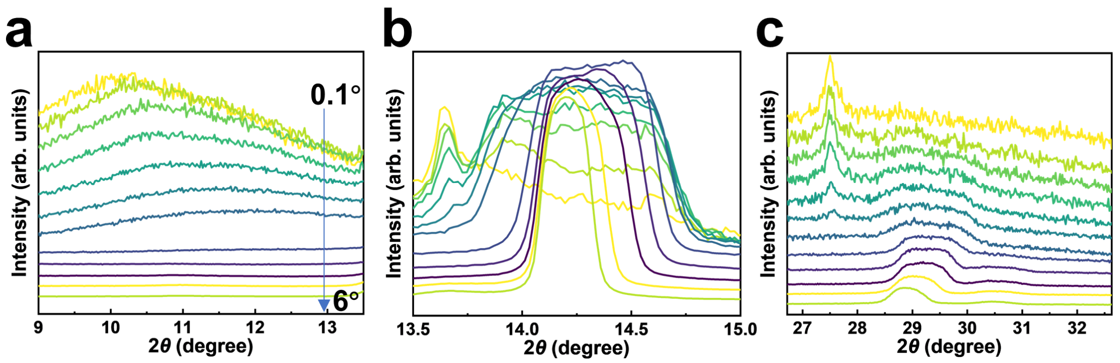

Supplementary Figure 2 | Grazing incident X-ray diffraction (GIXRD) patterns at different degrees. a, **b**, **c**, Partial magnification of the X-ray diffraction (XRD) patterns ranging from 9° to 13.5° (**a**), 13.5° to 15° (**b**), and 26.7° to 32.6° (**c**) shown in Fig. 1f.

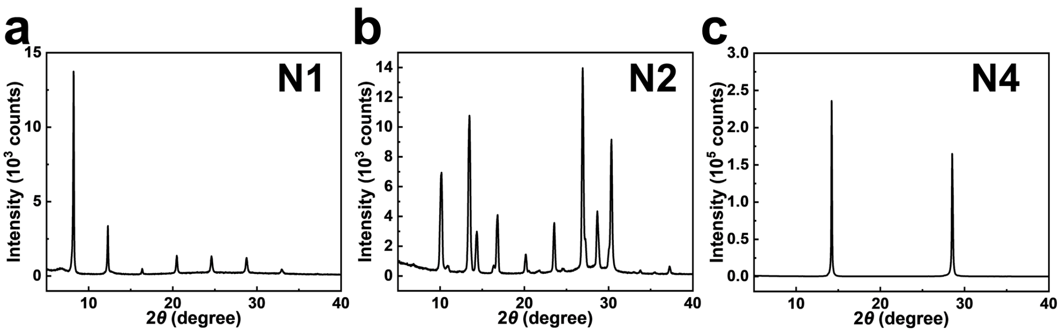

Supplementary Figure 3 | XRD patterns of 2D perovskites. a, **b**, **c**, XRD patterns for $(tBBA)_2PbI_4$ (N1) (**a**), $(tBBA)_2MAPb_2I_7$ (N2) (**b**), and $(tBBA)_2MA_3Pb_4I_{13}$ (N4) (**c**).

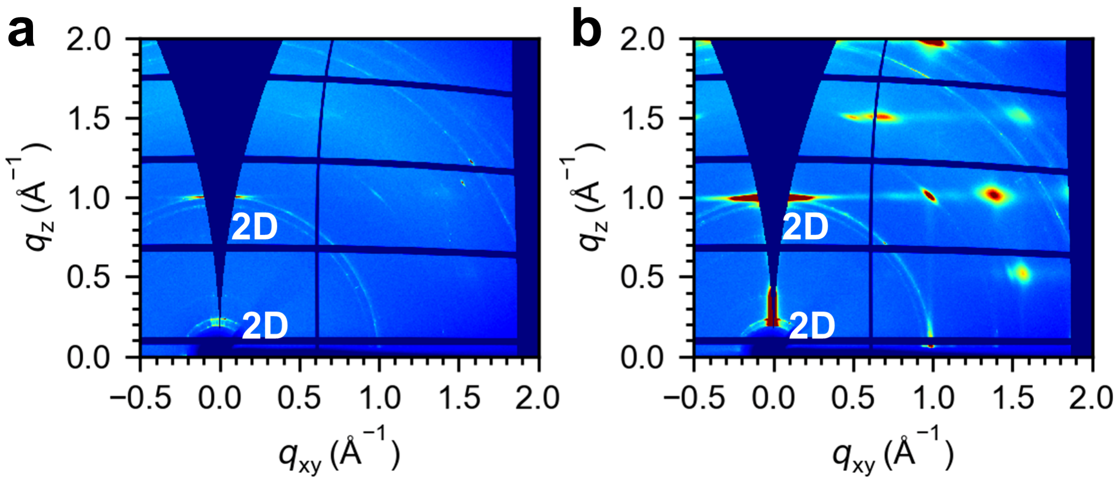

Supplementary Figure 4 | Grazing incidence wide angle X-ray scattering (GIWAXS) patterns of N3 perovksite. a, **b**, GIWAXS patterns of the N3 perovskite film based on tBBAI at grazing angles of 0.1° (**a**) and 0.5° (**b**).

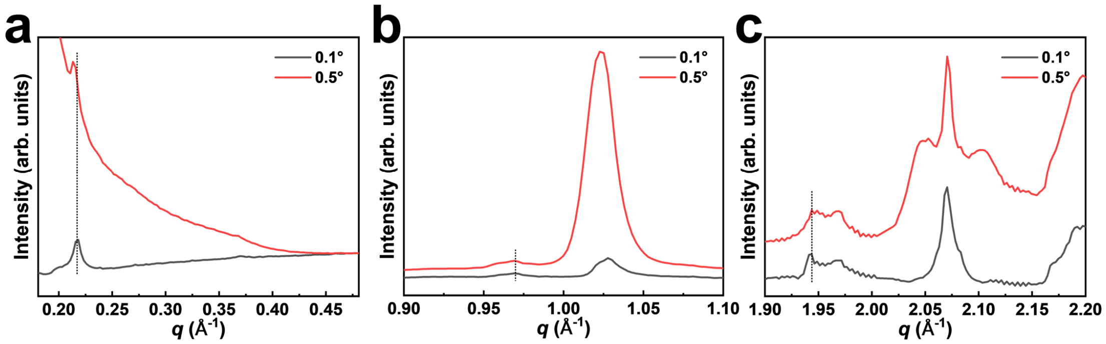

Supplementary Figure 5 | XRD patterns extracted from GIWAXS. a, **b**, **c**, Polar intensity profiles averaged along with the rings calculated from GIWAXS by Fit2D software.

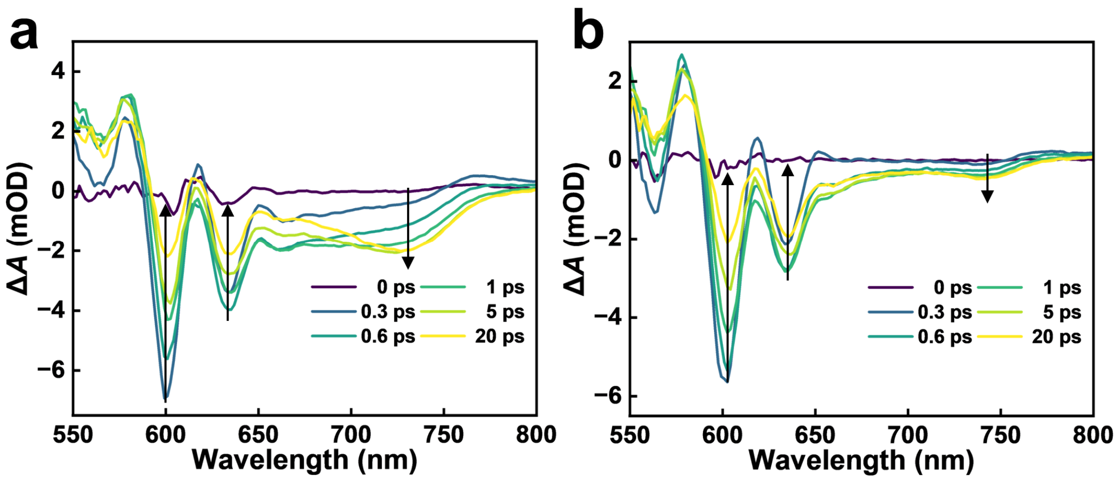

Supplementary Figure 6 | Transient absorption (TA) spectra. a, **b**, TA spectra recorded at different times from the perovskite (**a**) and glass (**b**) sides for a typical $(tBBA)_2MA_2Pb_3I_{10}$ 2D perovskite film.

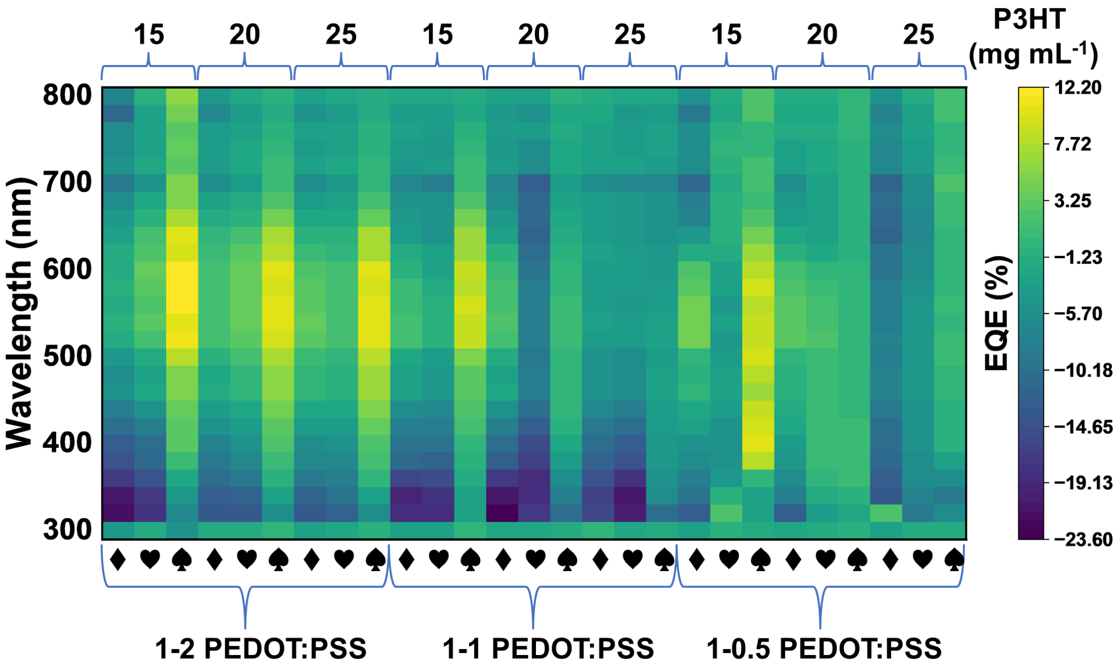

Supplementary Figure 7 | External quantum efficiency (EQE) of devices with different parameters. EQEs of the devices fabricated using different perovskite, P3HT, and PEDOT:PSS layer thicknesses. 1−*x* PEDOT:PSS, where *x* represents the ratio of PEDOT:PSS diluted in water; black rhombus-, heart-, and spade-shaped icons represent perovskite precursor concentrations of 1.0, 0.9, and 0.8 M, respectively. The large photoresponse at approximately 350 nm is from the absorption of the PEDOT:PSS layer.

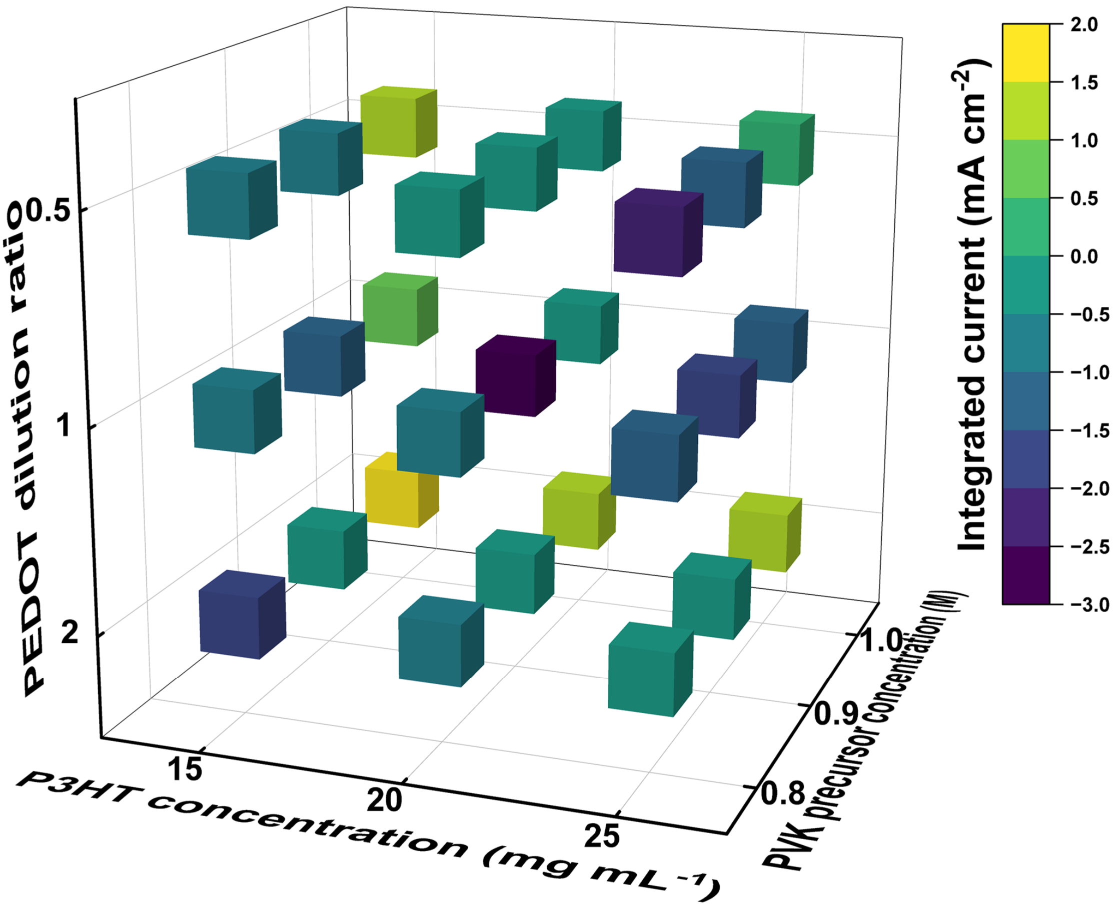

Supplementary Figure 8 | Integrated current of devices with different parameters. Integrated currents measured for the devices illuminated under AM 1.5G corresponding to the EQEs shown in Supplementary Fig. 5.

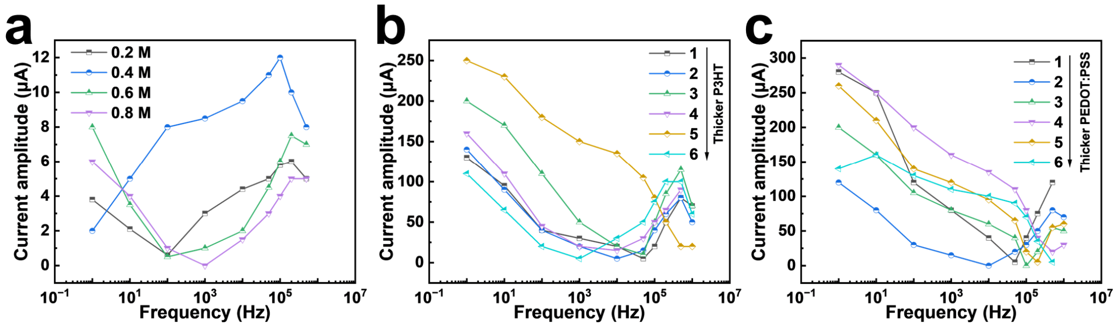

Supplementary Figure 9 | Photoresponse of devices with different parameters. a, b, c, Photocurrents measured for the devices fabricated using different thicknesses of perovskite (**a**), P3HT (**b**), and PEDOT:PSS (**c**) layers.

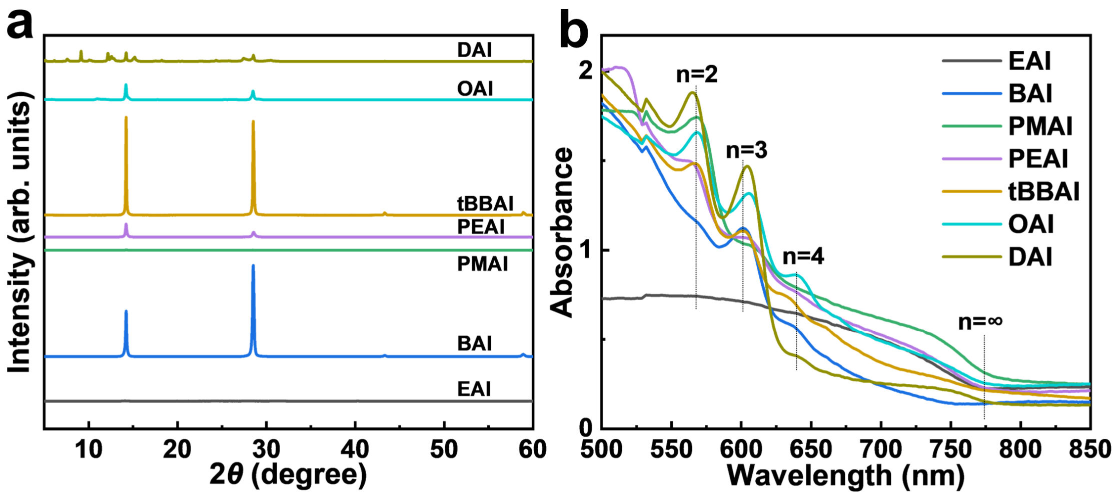

Supplementary Figure 10 | XRD and absorption spectra of 2D perovskites with different ammonium salts. a, **b**, XRD patterns (**a**) and absorption spectra (**b**) for the 2D perovskite films prepared using different 2D ammonium salts.

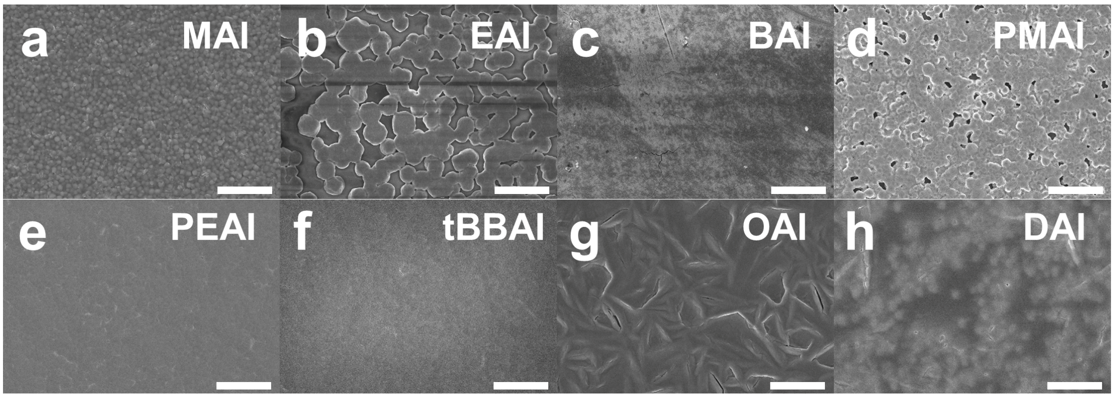

Supplementary Figure 11 | Scanning electron microscope (SEM) images of 2D perovskites with different ammonium salts. a-h, SEM images of the 2D perovskite films prepared using different ammonium salts for MAI (**a**), EAI (**b**), BAI (**c**), PMAI (**d**), PEAI (**e**), tBBAI (**f**), OAI (**g**), and DAI (**h**). The scale bar is 5 μm.

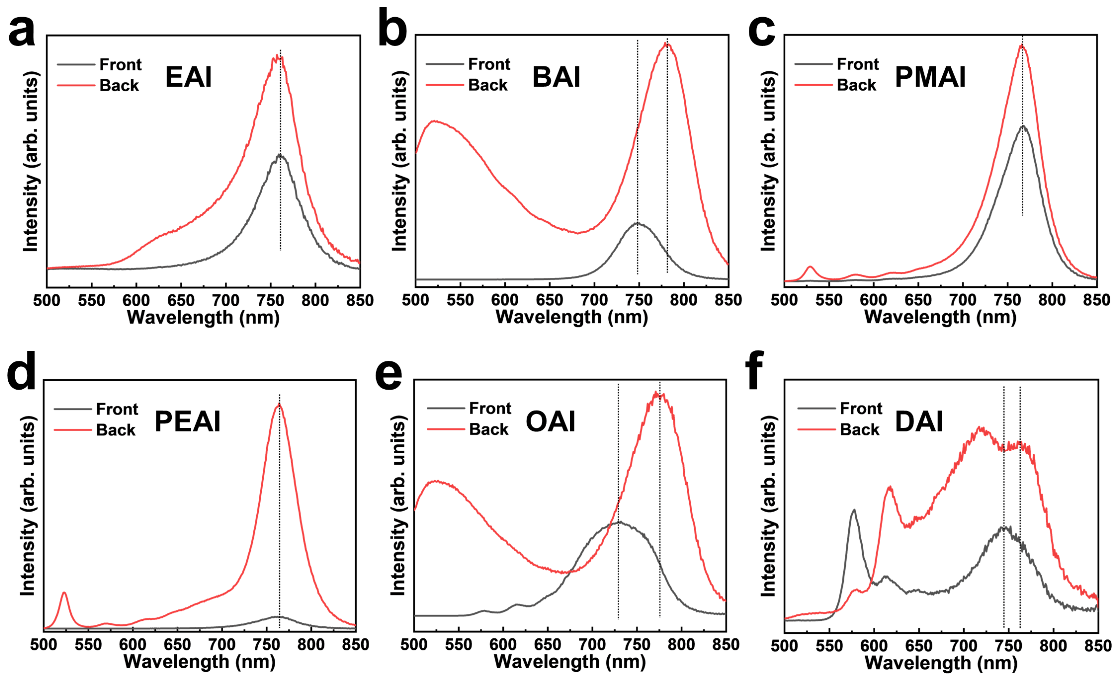

Supplementary Figure 12 | Photoluminescence (PL) spectra of 2D perovskites with different ammonium salts. a-f, PL spectra for the 2D perovskite films prepared using different 2D ammonium salts on glass substrates for EAI (**a**), BAI (**b**), PMAI (**c**), PEAI (**d**), OAI (**e**), and DAI (**f**).

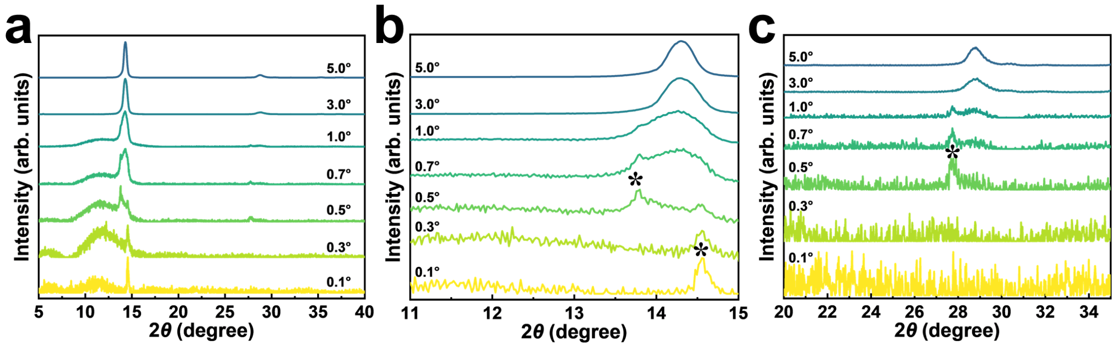

Supplementary Figure 13 | GIXRD patterns of 2D perovskites based on PEAI. a, b, c, Enlarged GIXRD patterns with grazing angles from 0.1° to 5° for the N3 perovskite based on PEAI. The black star represents the 2D perovskite phase.

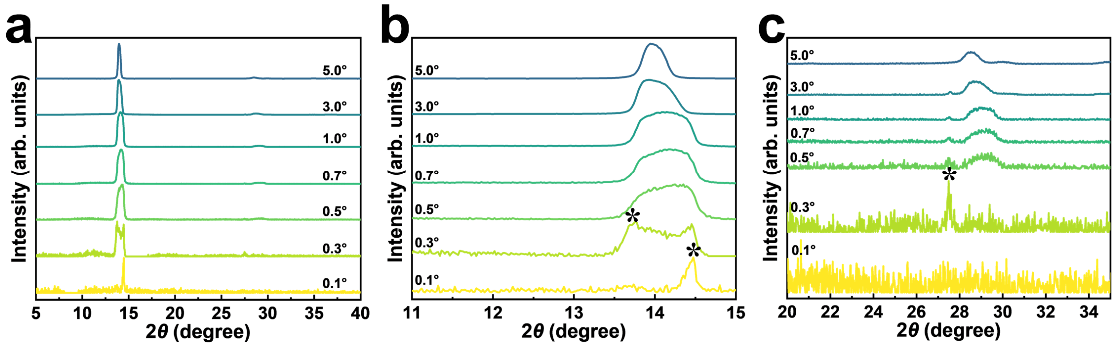

Supplementary Figure 14 | GIXRD patterns of 2D perovskites based on BAI. a, **b**, **c**, Enlarged GIXRD patterns with grazing angles from 0.1° to 5° for the N3 perovskite based on BAI. The black star represents the 2D perovskite phase.

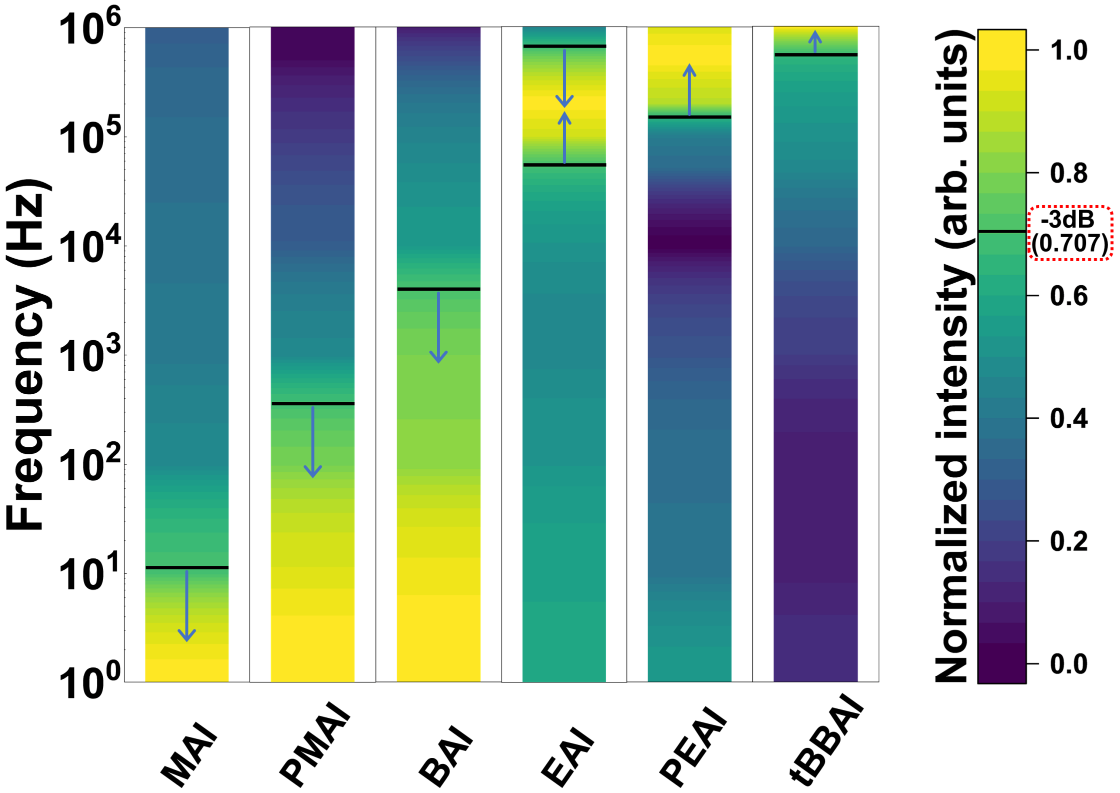

Supplementary Figure 15 | Photoresponse of devices based on different perovskites. Normalized current amplitude of the devices based on different 2D ammonium salts at different frequencies.

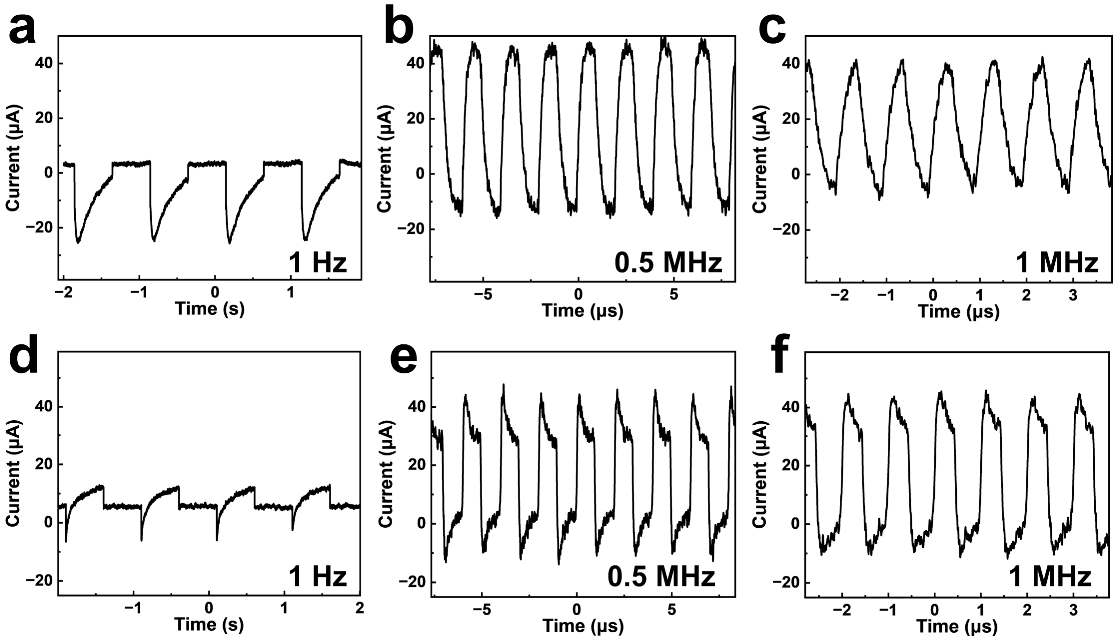

Supplementary Figure 16 | Photoresponse of devices based on PEAI and tBBAI perovskites. **a**, **b**, **c**, Photoresponse measured at different frequencies for the PEAI-based back-to-back-structured devices at 1 Hz (**a**), 0.5 MHz (**b**), and 1 MHz (**c**). **d**, **e**, **f**, Photoresponse measured at different frequencies for tBBAI-based back-to-back-structured devices at 1 Hz (**d**), 0.5 MHz (**e**), and 1 MHz (**f**).

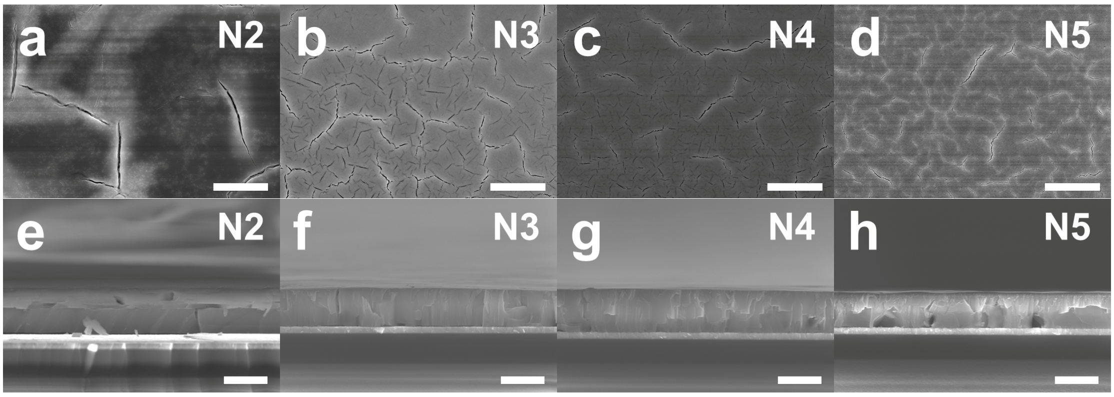

Supplementary Figure 17 | SEM images of tBBAI-based perovskites with different N values. a-d, Top-view SEM images of the tBBAI-based N2 (**a**), N3 (**b**), N4 (**c**), and N5 (**d**) perovskite films. The scale bar is 5 μm. **e-h**, Cross-sectional SEM images of the tBBAI-based N2 (**e**), N3 (**f**), N4 (**g**), and N5 (**h**) perovskite films. The scale bar is 1 μm.

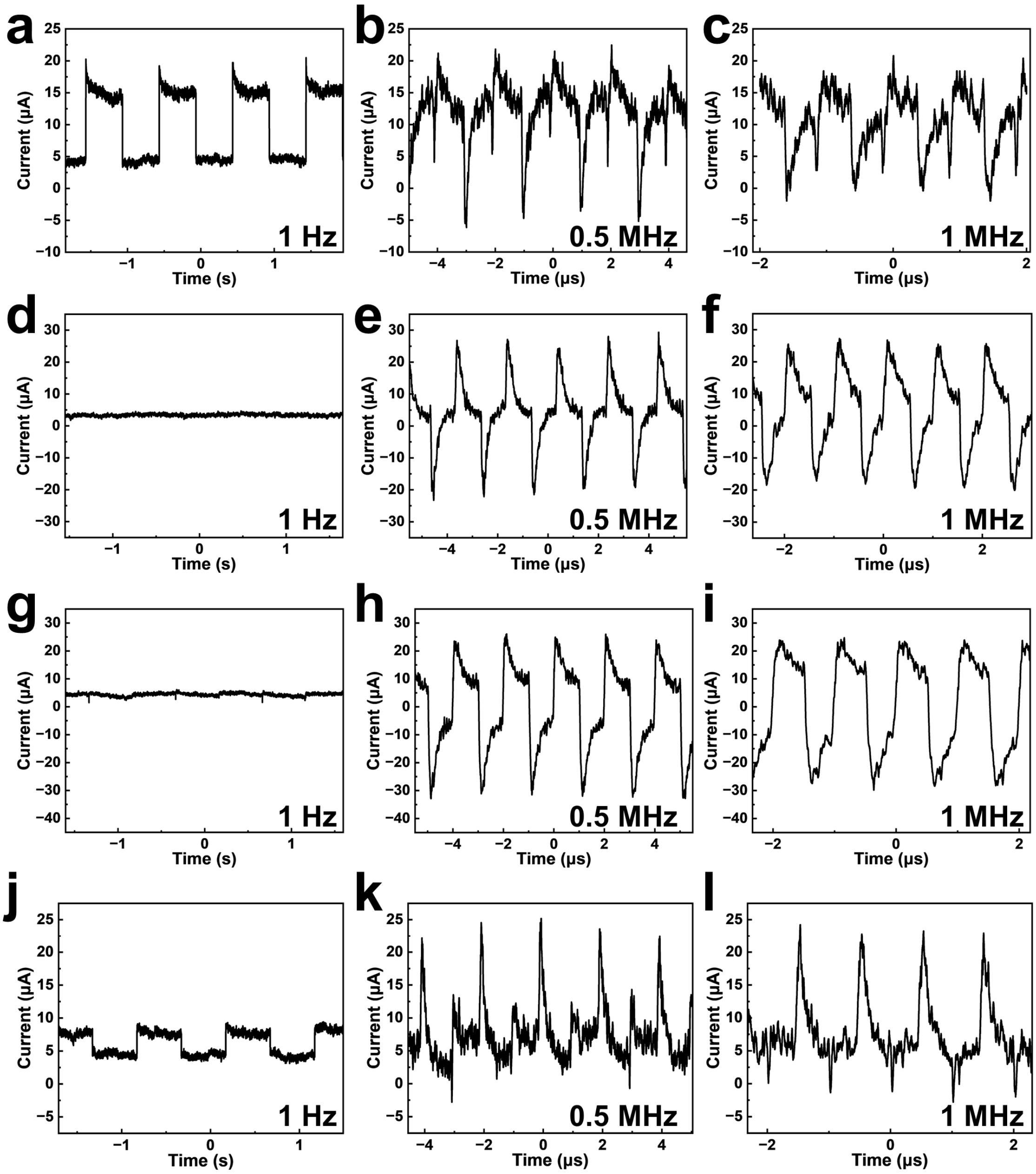

Supplementary Figure 18 | Photoresponse of tBBAI-based perovskites with different N values. a-c, Photoresponse measured at 1 Hz (**a**), 0.5 MHz (**b**), and 1 MHz (**c**) for the N2 perovskite-based device. **d-f**, Photoresponse measured at 1 Hz (**d**), 0.5 MHz (**e**), and 1 MHz (**f**) for the N3 perovskite-based device. **g-i**, Photoresponse measured at 1 Hz (**g**), 0.5 MHz (**h**), and 1 MHz (**i**) for the N4 perovskite-based device. **j-l**, Photoresponse measured at 1 Hz (**j**), 0.5 MHz (**k**), and 1 MHz (**l**) for the N5 perovskite-based device.

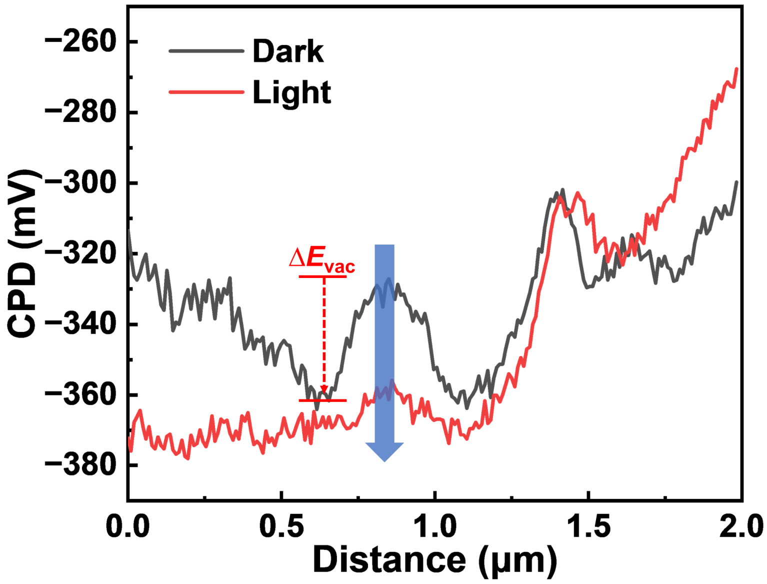

Supplementary Figure 19 | Contact potential differences (CPDs). CPDs measured under dark and light conditions for the cross-sectional Ag/P3HT/perovskite/PEDOT:PSS/ITO device.

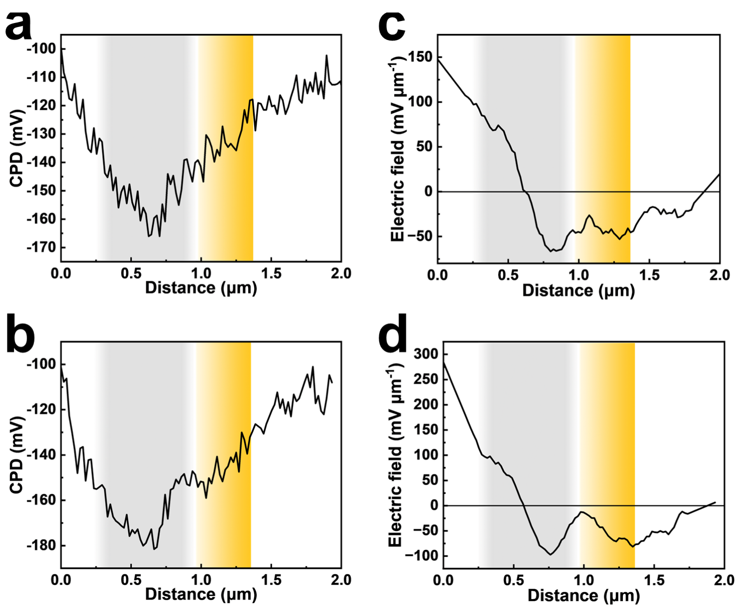

Supplementary Figure 20 | Kelvin probe force microscopy (KPFM) test of pure N3 perovskite. a, **b**, CPDs measured under dark (**a**) and light (**b**) conditions for the N3 perovskite film deposited on an ITO substrate. **c**, **d**, Electric field difference under dark (**c**) and light (**d**) conditions obtained by calculating the first derivatives of **a** and **b**. **e**, **f**, Charge-density distribution profiles under dark (**e**) and light (**f**) conditions obtained by calculating the second derivatives of **a** and **b**.

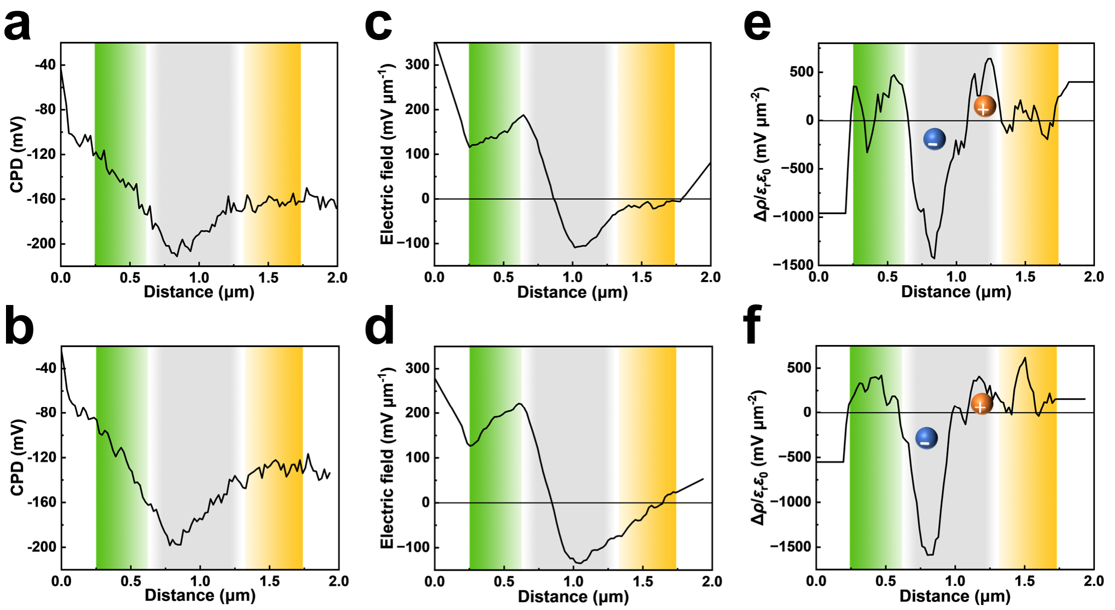

Supplementary Figure 21 | KPFM test of PEAI-based device. a, **b**, CPDs measured under dark (**a**) and light (**b**) conditions for the PEAI-based device. **c**, **d**, Electric field difference under dark (**c**) and light (**d**) conditions obtained by calculating the first derivatives of **a** and **b**. **e**, **f**, Charge-density distribution profiles under dark (**e**) and light (**f**) conditions obtained by calculating the second derivatives of **a** and **b**.

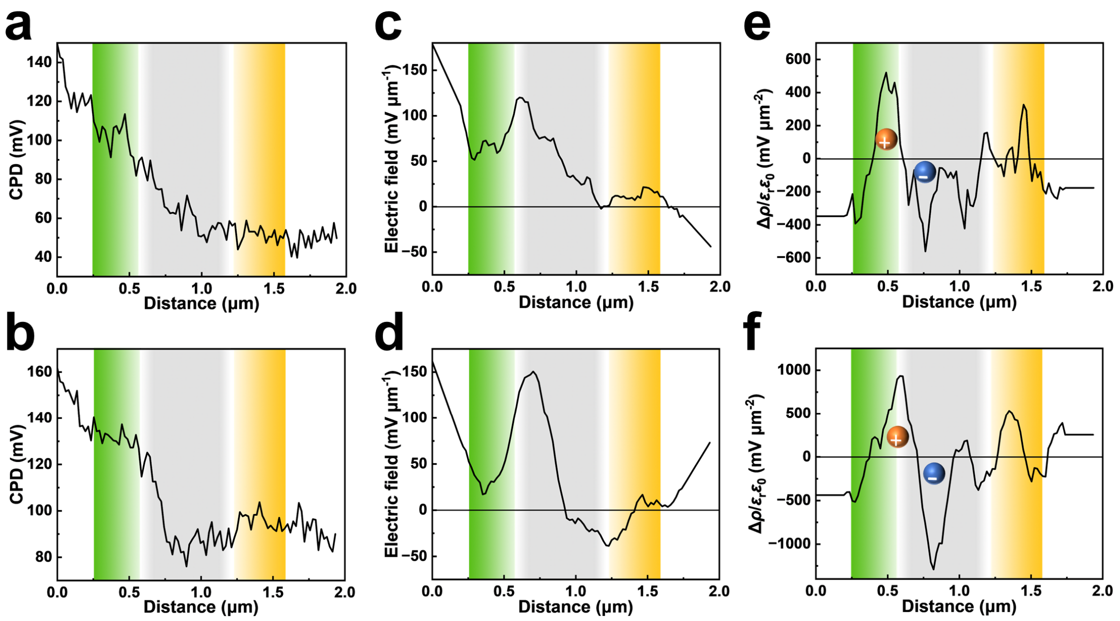

Supplementary Figure 22 | KPFM test of MAI-based device. a, **b**, CPDs measured under dark (**a**) and light (**b**) conditions for the $MAPbI_3$-based device. **c**, **d**, Electric field difference under dark (**c**) and light (**d**) conditions obtained by calculating the first derivatives of **a** and **b**. **e**, **f**, Charge-density distribution profiles under dark (**e**) and light (**f**) conditions obtained by calculating the second derivatives of **a** and **b**.

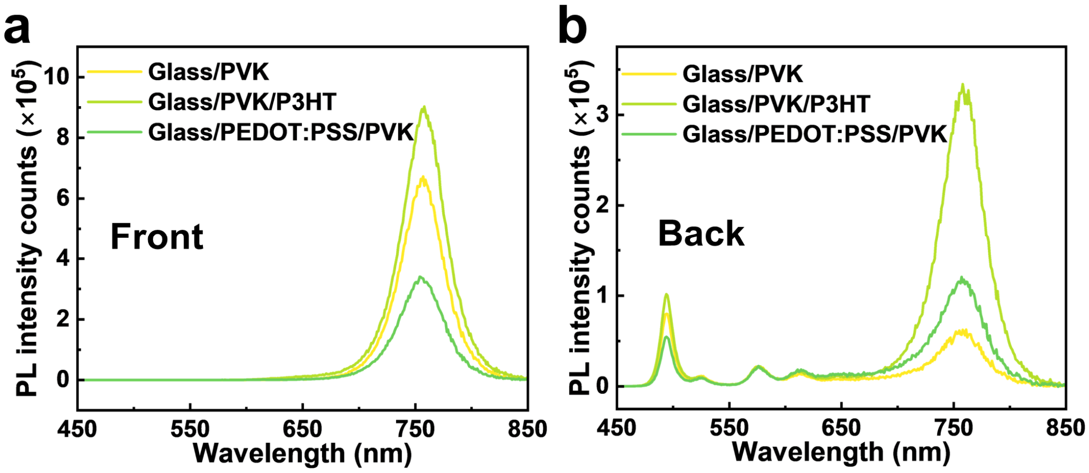

Supplementary Figure 23 | PL measurement. a, **b**, PL spectra of a single perovskite film and two single-HTL/perovskite films from the front (**a**) and back (**b**) sides.

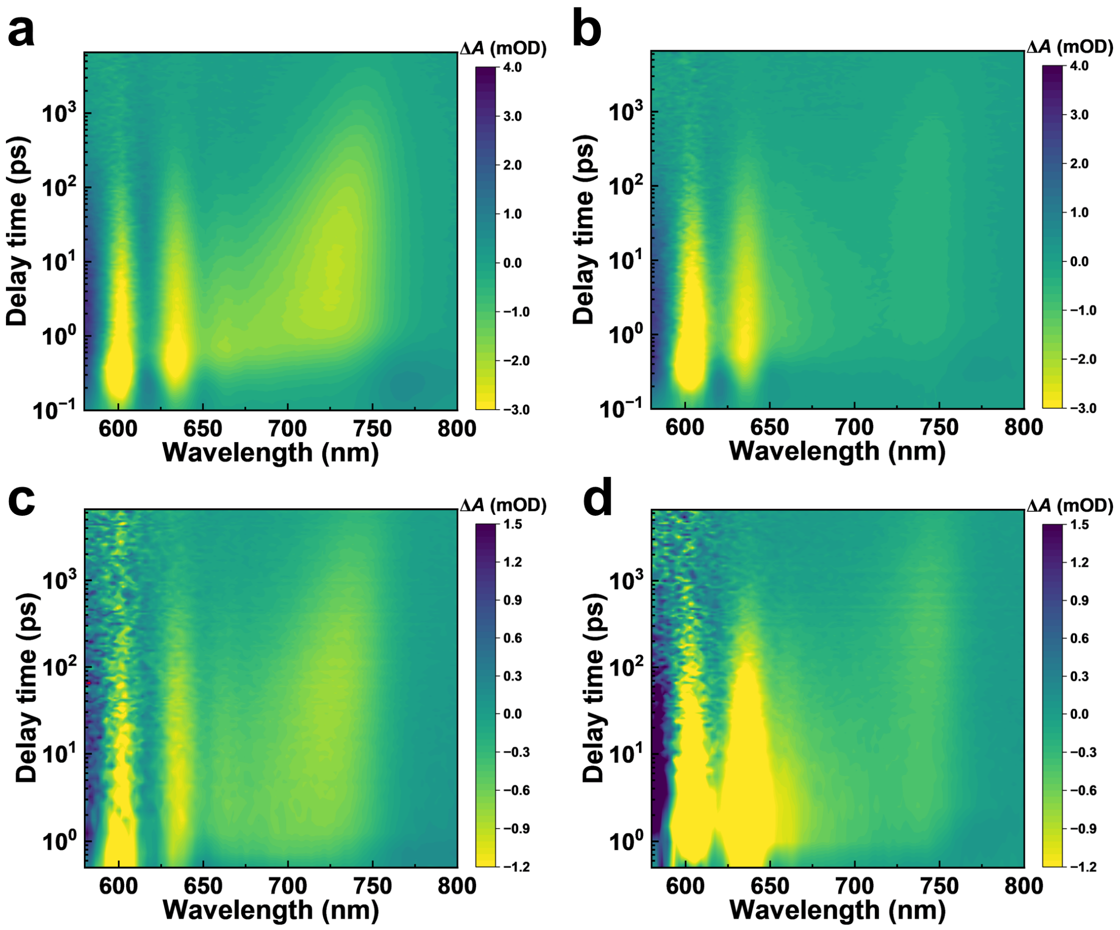

Supplementary Figure 24 | TA mapping. a-d, Time-wavelength-dependent TA colour maps of the N3 perovskite/PEDOT:PSS (**a**, **b**) and P3HT/perovskite (**c**, **d**) deposited on glass from the perovskite side and glass side.

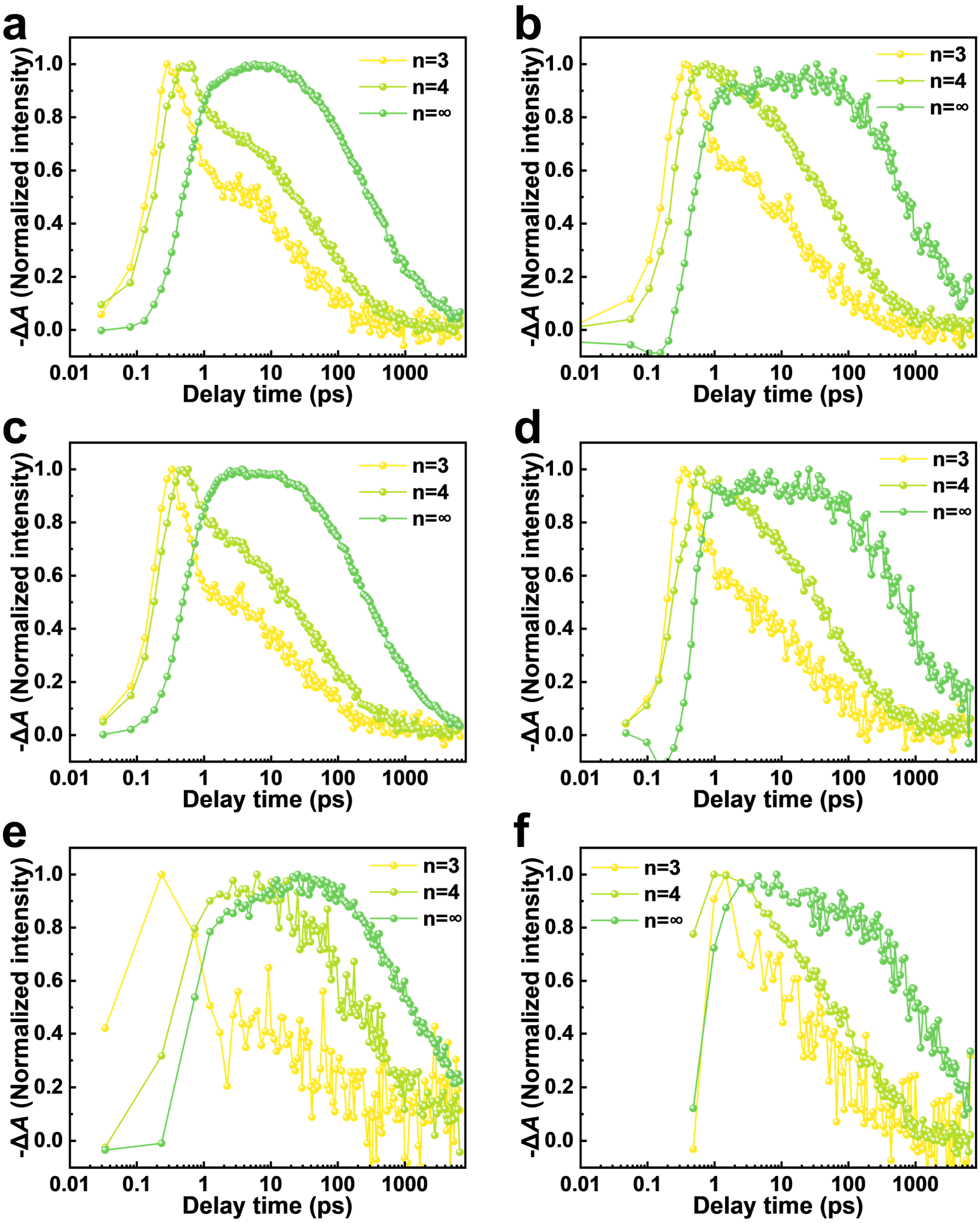

Supplementary Figure 25 | TA spectra. a, **b**, Normalized TA dynamics for bleaching recoveries of the N3 perovskite/glass from the front (**a**) and back (**b**) sides. **c**, **d**, Normalized TA dynamics for bleaching recoveries of the N3 perovskite/PEDOT:PSS/glass from the front (**c**) and back (**d**) sides. **e**, **f**, Normalized TA dynamics for bleaching recoveries of the P3HT/N3 perovskite/glass from the front (**e**) and back (**f**) sides.

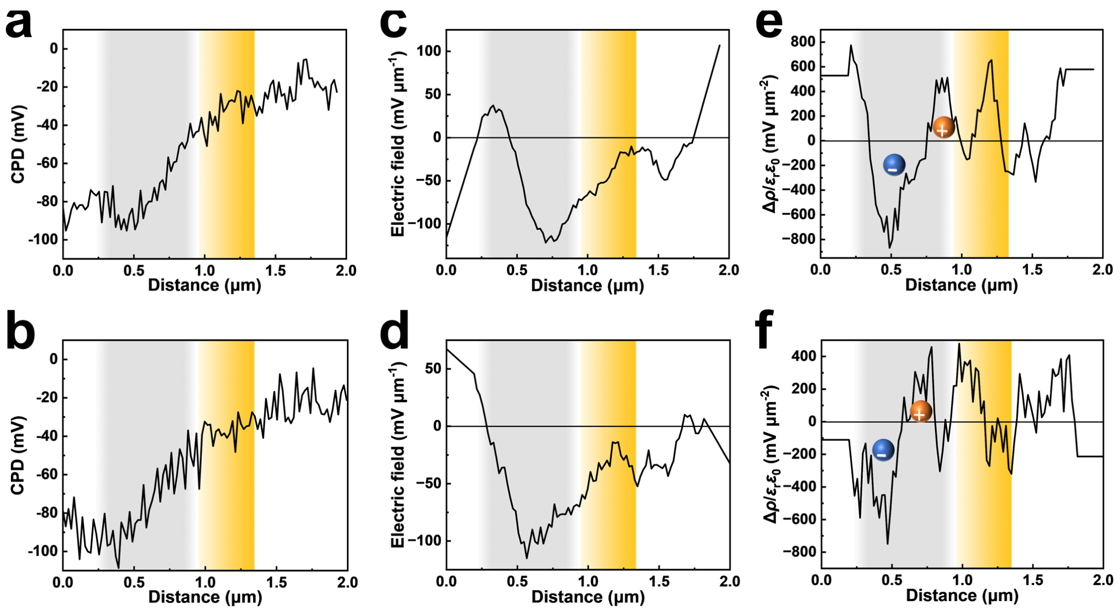

Supplementary Figure 26 | KPFM test of tBBAI-based device without P3HT. a, b, CPDs measured under dark (**a**) and light (**b**) conditions for the Cu/N3 perovskite/PEDOT:PSS/ITO device. **c**, **d**, Electric field difference under dark (**c**) and light (**d**) conditions obtained by calculating the first derivatives of **a** and **b**. **e**, **f**, Charge-density distribution profiles under dark (**e**) and light (**f**) conditions obtained by calculating the second derivatives of **a** and **b**.

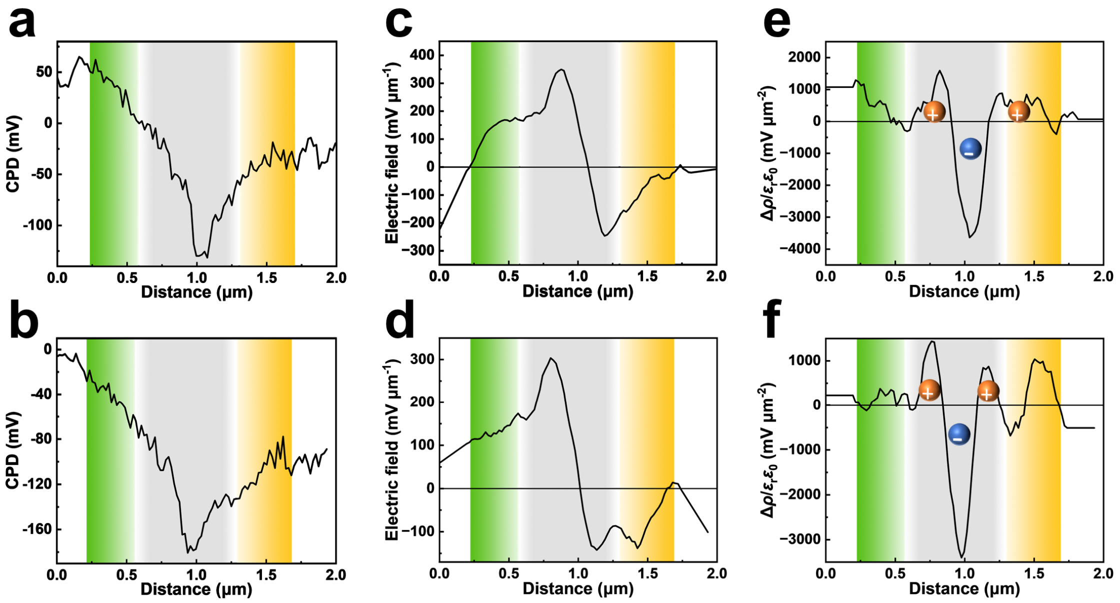

Supplementary Figure 27 | KPFM test of tBBAI-based device without PEDOT:PSS. a, **b**, CPDs measured under dark (**a**) and light (**b**) conditions for the Ag/P3HT/N3 perovskite/ITO device. **c**, **d**, Electric field difference under dark (**c**) and light (**d**) conditions obtained by calculating the first derivatives of **a** and **b**. **e**, **f**, Charge-density distribution profiles under dark (**e**) and light (**f**) conditions obtained by calculating the second derivatives of **a** and **b**.

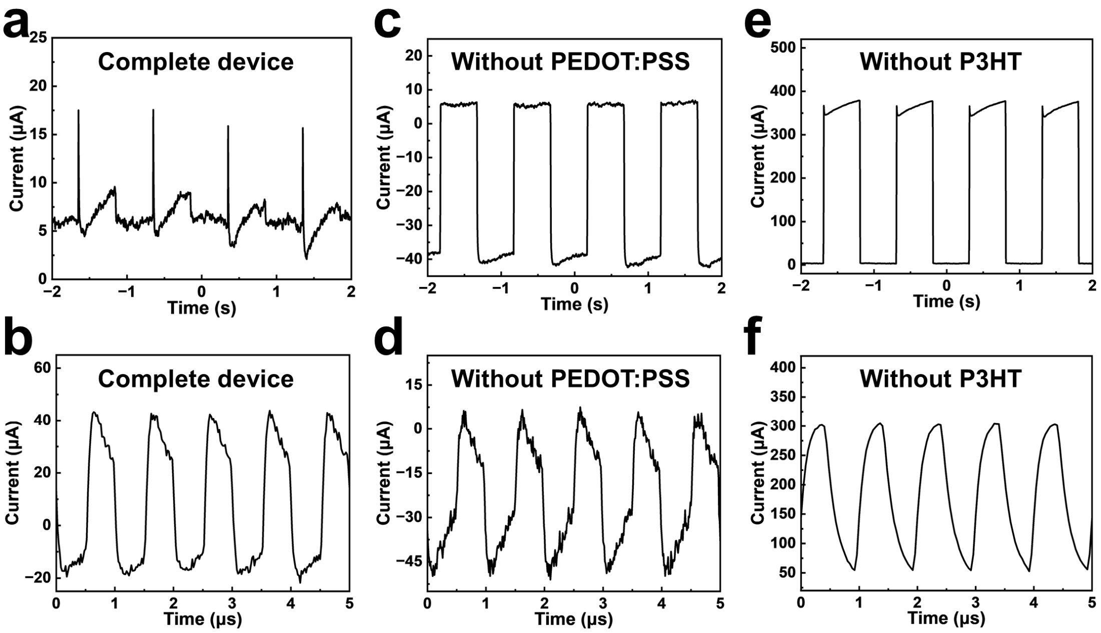

Supplementary Figure 28 | Photoresponse of tBBAI-based full and half devices. a, b, Photoresponse of the Ag/P3HT/N3 perovskite/PEDOT:PSS/ITO device measured at 1 Hz (**a**) and 1 MHz (**b**). **c**, **d**, Photoresponse of the Ag/P3HT/N3 perovskite/ITO device measured at 1 Hz (**c**) and 1 MHz (**d**). **e**, **f**, Cu/N3 perovskite/PEDOT:PSS/ITO device measured at 1 Hz (**e**) and 1 MHz (**f**).

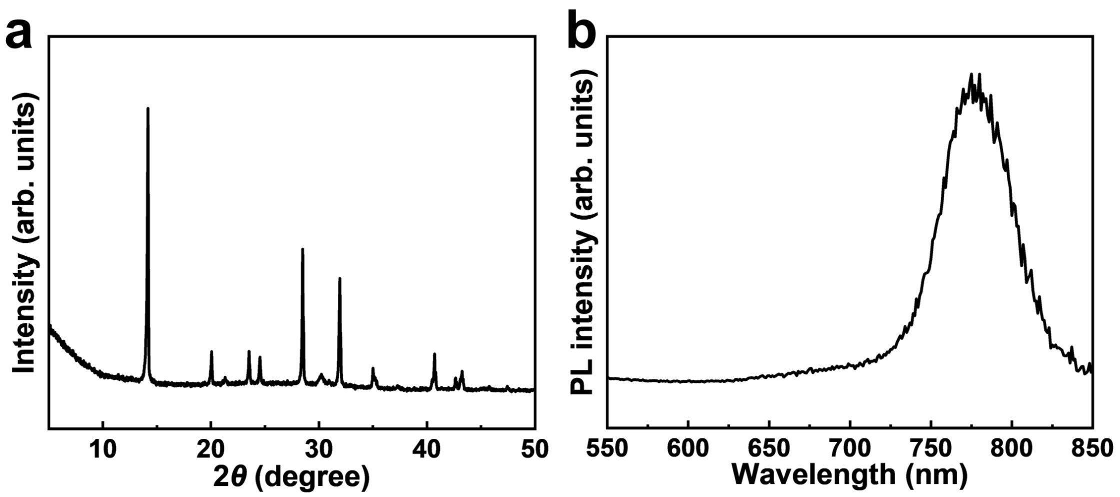

Supplementary Figure 29 | Characterization of $MAPbI_3$. a, XRD pattern of $MAPbI_3$. **b**, PL spectrum of $MAPbI_3$ on a glass substrate.

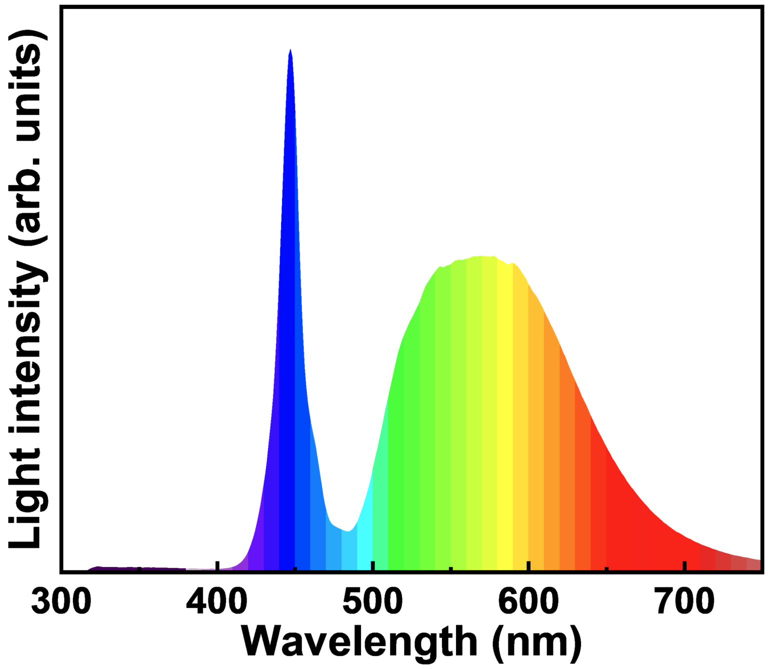

Supplementary Figure 30 | Light emitting diode (LED) spectrum. Interference spectrum generated using an LED lamp.

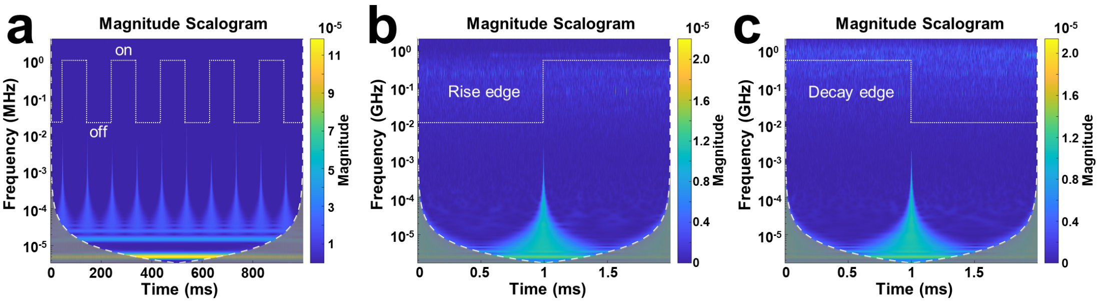

Supplementary Figure 31 | Wavelet transform of square wave signal. a, Frequency distribution of a 5 Hz square wave processed with the wavelet transformation. **b**, **c**, Frequency distribution of the rise edge (**b**) and decay edge (**c**) processed with the wavelet transformation at a sample rate of 5 GS/s.
