# Supplementary material for: Frequency-selective perovskite photodetector for anti-interference optical communications
Source: Nat Commun. 2024 Mar 7;15:2066. doi: 10.1038/s41467-024-46468-5 (PMC10920912; doi:10.1038/s41467-024-46468-5)
Supplement: Supplementary file 3 — Description of Additional Supplementary Files [file 41467_2024_46468_MOESM3_ESM.pdf]

Description of Additional Supplementary Information

Supplementary Movie 1:

Device with structure of ITO/SnO_2/$MAPbI_3$/Spiro-OMeTAD/Ag was selected as n-i(3D)-p type control device. In the presence of LED interference of only 55.1 mW cm^{-2}, the signals show distortion.

Supplementary Movie 2:

Device with structure of ITO/PEDOT:PSS/N3 perovskite/P3HT/Ag was selected as the target device to transmit character signals. It can transmit characters accurately under the LED interference of 454 mW cm^{-2}.

Supplementary Movie 3:

Device with structure of ITO/PEDOT:PSS/$MAPbI_3$/P3HT/Ag was selected as p-i(3D)-p type control device. The p-i(3D)-p-type device could barely withstand the interference of 55.1 mW cm^{-2}, indicating the necessity of the V-shaped built-in electric field.

Supplementary Movie 4:

Device with structure of ITO/PEDOT:PSS/N3 perovskite/PCBM/Ag was selected as p-i(2D-3D-2D)-n type control device. This is akin to the n-i(3D)-p type control device, which also lacks any anti-interference capabilities. This underscores the importance of incorporating double hole transport layers.

Supplementary Movie 5:

The target device was utilized to showcase the transmission of video signals under varying intensities of LED interference. It is capable of continuously transmitting video signals until the intensity of the interference light reaches 910 mW cm^{-2}.

Supplementary Movie 6:

The target device was employed to exhibit video transmission under LED interference that was modulated with sine waves. A continuous and stable video transmission has been accomplished under such LED interference.

Supplementary Movie 7:

A commercial high-speed silicon photodetector (model: S6968, Hamamatsu) was employed as a reference for video signal transmission. While it is capable of delivering clear video transmission, even minor external interference (below 55.1 mW cm^{-2}) can lead to instantaneous signal loss.
